# Tuned Inhibitory Responses in Binocular Natural Images

**DOI:** 10.1068/ig8

**Published:** 2013-10-01

**Authors:** R Goutcher, D W Hunter, P B Hibbard

**Affiliations:** University of Stirling, UK; University of St. Andrews, UK; University of Essex, UK

## Abstract

Binocular neurons in primary visual cortex are typically categorised as either Near, Far, Tuned Excitatory (TE) or Tuned Inhibitory (TI) cells (Poggio & Fischer, 1977, J. Neurophysiol., 40, 1392-1405). Both TI and TE responses may be approximated through differing arrangements of the binocular energy model (Ohzawa, De Angelis & Freeman, 1990, Science, 249, 1037-1041), with TE neurons responding to instances of binocular correlation, and TI neurons responding to a lack of correlation. Differences in the inter-ocular phase or position of receptive fields allow model TE cells to be used for the measurement of binocular disparity. Previous research (Hibbard, 2008, Vision Research, 48, 1427-1439) has examined the responses of these disparity tuned TE neurons to binocular natural images. However, the responses of TI neurons to such images are not clear. We examined these TI responses for a set of binocular natural images, and compared them to responses for random-dot stereograms (RDSs). TI neuron responses were modelled by placing left and right eye receptive fields in anti-phase arrangement. In addition, TI responses were normalised by monocular energy. This provides a response with a range of ±1, with a value of +1 indicating that two image regions are anti-correlated. TI responses were generally negative for both RDSs and natural images. For binocular natural images, positive TI responses were found for a small proportion of units. Typically, positive TI responses were found when monocular energy was low, occurred across all orientation channels, and increased in prevalence at lower spatial frequencies. Positive responses were clustered around object edges, suggesting a functional role for TI neurons in the detection of depth boundaries.

